# Augmentation of neovascularization in murine hindlimb ischemia by combined therapy with simvastatin and bone marrow-derived mesenchymal stem cells transplantation

**DOI:** 10.1186/1423-0127-17-75

**Published:** 2010-09-17

**Authors:** Yong Li, Dingguo Zhang, Yuqing Zhang, Guoping He, Fumin Zhang

**Affiliations:** 1 Department of Cardiology, the First Affiliated Hospital of Nanjing Medical University, 210029, R.R. China; 2 Department of Cardiology, the Affiliated Wujin Hospital of Jiangsu University, 213002, P.R. China; 3 The Research Center for Bone And Stem Cells, Nanjing Medical University, 210029, P.R. China; 4 Department of Cardiology, the Affiliated Jiangning Hospital of Nanjing Medical University, 211100, P.R. China

## Abstract

**Objectives:**

We postulated that combining high-dose simvastatin with bone marrow derived-mesenchymal stem cells (MSCs) delivery may give better prognosis in a mouse hindlimb ischemia model.

**Methods:**

Mouse hindlimb ischemia model was established by ligating the right femoral artery. Animals were grouped (n = 10) to receive local injection of saline without cells (control and simvastatin groups) or with 5 × 10^6 ^MSCs (MSCs group).Animals received either simvastatin (20 mg/kg/d, simvastatin and combination groups) or saline(control and MSCs group) gavages for continual 21 days. The blood flow was assessed by laser Doppler imaging at day 0,10 and 21 after surgery, respectively. Ischemic muscle was harvested for immunohistological assessments and for VEGF protein detection using western blot assay at 21 days post-surgery. In vitro, MSCs viability was measured by MTT and flow cytometry following culture in serum-free medium for 24 h with or without simvastatin. Release of VEGF by MSCs incubated with different doses of simvastatin was assayed using ELISA.

**Results:**

Combined treatment with simvastatin and MSCs induced a significant improvement in blood reperfusion, a notable increase in capillary density, a highest level of VEGF protein and a significant decrease in muscle cell apoptosis compared with other groups. In vitro, simvastatin inhibited MSCs apoptosis and increased VEGF release by MSCs.

**Conclusions:**

Combination therapy with high-dose simvastatin and bone marrow-derived MSCs would augment functional neovascularization in a mouse model of hindlimb ischemia.

## Introduction

Peripheral arterial disease (PAD) is one of the most common clinical manifestations of atherosclerosis, which affects a significant number of individuals. It represents an important cause of disability and is associated with elevated cardiovascular morbidity and mortality[1]. Treatment of PAD includes anticoagulants and antiplatelet drugs, percutaneous transluminal angioplasty, and bypass surgery. However, the prognosis for patients with PAD still remains poor, and amputation of the lower extremities is often required [2]. Several types of stem cells have been used for therapeutic neovascularization, including the bone marrow-derived mesenchymal stem cells (MSCs), which have attracted a great attention from investigators because of their plasticity and availability[3]. These cells mediate their therapeutic effects by homing to and integrating into injured tissues, differentiating into endothelial cells, and/or producing paracrine growth factors. However, recent studies have shown that patients with PAD are often coincident with cardiovascular risk factors, such as aging, diabetes mellitus, which reduce the availability of progenitor cells and impair their function to varying degrees[4-6], likely limiting the efficiency of stem cell therapy. Therefore, optimization of strategies to improve the therapeutic potential of cell therapy needs to be developed to augment application of this technology for patients with cardiovascular diseases.

Statins are 3-hydroxy-3-methyl-glutaryl-CoA reductase inhibitors and are primarily used to lower circulating cholesterol levels. In addition, studies have revealed statin's pleiotropic effects, such as the protection of endothelial function, increased nitric oxide bioavailability, antioxidant effects, anti-inflammatory reaction, and stabilization of atherosclerotic plaques[7,8]. Recent studies have demonstrated that statins could protect against ischemic injury of the heart [9]and stimulate angiogenesis in ischemic limbs of normocholesterolemic animals [10]. However, both in vitro and in vivo studies have suggested a biphasic and dose-dependent effect of statins on angiogenesis [11]. Yang demonstrated that low-dose simvastatin could enhance the therapeutic effects of bone marrow cells in pig's acute myocardial infarction model [12]. Whereas, some studies indicated that high-dose statins could also enhance angiogenesis in vivo [13]. Accordingly, we investigated whether the combination therapy with high-dose simvastatin administration and MSCs transplantation could augment functional neovascularization in a mouse model of hind limb ischemia.

## Materials and methods

### Animals

Adult male Sprague-Dawley rats (80-100 g) were purchased from Slac company (Shanghai, China).Adult female C57BL/6J mice (8 weeks, 20-25 g) were provided by the Model Animal Research Center of Nanjing University (Nanjing, China). All animal experimental protocols were approved by the Animal Care and Use Committee of Nanjing Medical University and were in compliance with Guidelines for the Care and Use of Laboratory Animals, as published by the National Academy Press (NIH Publication No. 85-23, revised 1996)

### Isolation, expansion and labeling of MSCs

Rat MSCs were isolated with a modified procedure as described previously [14]. In brief, Sprague-Dawley rats were sacrificed by cervical dislocation. Femora and tibia were aseptically harvested. Whole marrow cells were obtained by flushing the bone marrow cavity with low glucose Dulbecco's Modified Eagle's Medium (L-DMEM, Hyclone, USA). Cells were centrifuged at 1000 × *g *for 5 minutes and the supernatant was removed. The cell pellet was then re-suspended with L-DMEM supplemented with 10% fetal bovine serum (FBS, Hyclone, USA), 100 U/ml penicillin (Gibco,USA), 100 U/ml streptomycin (Gibco,USA), and incubated at 37°C in a 5% CO_2 _atmosphere. After 24 hours, non-adherent cells in suspension were discarded and culture media was changed every three or four days thereafter. When MSCs reached 70%-80% of confluence, they were trypsinized by the addition of 0.25% trypsin-EDTA (Sigma-Aldrich, USA), and then re-plated in culture flasks. Cells between 3^rd ^and 6^th ^passage were utilized for experiment.

### Mouse Model of Unilateral Hindlimb Ischemia

Unilateral hindlimb ischemia was created in 8-week-old female C57BL/6J mice as described previously [15,16]. Briefly, mice were anesthetized with pentobarbital (50 mg/kg, intraperitoneally) and the right femoral artery was dissected free along its entire length. All branches were ligated and excised. The left hindlimb was kept intact and used as the nonischemic limb.

### Simvastatin administration and MSCs transplantation

Simvastatin administration and MSCs transplantation were performed immediately after hindlimb ischemia was created. Simvastatin (20 mg/kg per day) or vehicle (saline) was administered every day by gavage for 21 days. MSCs (5 × 10^6 ^cells/50 μl per mouse) or 50 μl saline was injected into the ischemic thigh muscle with a 26-gauge needle at five different points. This protocol resulted in the creation of four groups (n = 10/group): (1) vehicle administration plus saline injection (control group), (2) simvastatin administration plus saline injection (simvastatin group), (3) vehicle administration plus MSCs transplantation (MSCs group), (4) simvastatin administration plus MSCs transplantation (combination group). Simvastatin was kindly donated by Merck & Co., Inc., USA. MSCs were labeled with 1,1'-dioctadecyl-3,3,3'3'-testramethylindo-carbocyanine perchlorate (DiI) before transplantation as described previously[17]. Briefly, 2 μg/ml DiI was added to cells suspension and incubated at 37°C for 5 minutes, then at 4°C for 15 minutes with occasional mixing. MSCs labeled with DiI were washed 3 times with PBS and then collected.

### Laser Doppler blood perfusion analysis

The ratio of ischemic/normal hindlimb blood flow was measured using laser Doppler blood perfusion imager (PeriScan PIM 3, Swenden) as described previously[15,16].Low to no flow was displayed as dark blue, whereas high blood flow was displayed as red to white. Previous study has demonstrated [16] that hindlimb blood flow was progressively augmented over the course of 14 days, ultimately reaching a plateau between 21 and 28 days in mouse hindlimb ischemia model. Therefore, at three predetermined time points (immediately after surgery, and on postoperative days 10 and 21), we performed 2 consecutive laser scanning over the same region of interest (legs and feet). The average flow of the ischemic and nonischemic legs was calculated on the basis of histograms of the colored pixels. To minimize variations due to ambient light, blood flow was expressed as the ischemic (right)/normal (left) limb flow ratio.

### Histological assessment for capillary density

Ischemic limb muscles were harvested at day 21 after treatment and embedded in optimal cutting temperature compound. Frozen tissue sections of 5 μm-thick were stained for alkaline phosphatase [18] to examine the capillary density. To ensure that the capillary densities were not overestimated as a consequence of myocyte atrophy or underestimated because of interstitial edema, the capillary/muscle fiber ratio was determined.

### Terminal deoxynucleotidy1 transferase-mediated dUTP nick end-labeling assay

The terminal deoxynucleotidy1 transferase-mediated dUTP nick end-labeling (TUNEL) assay was performed to determine apoptotic activity in hindlimb ischemic tissues using an In Situ Cell Death Detection Kit (Roche, Germany) according to the manufacturer's instructions. Cells in which the nucleus was stained brown were defined as TUNEL-positive and the percentage of apoptotic cells per total number of cells was determined by two independent blinded investigators.

### Western blot analysis for the expression of VEGF protein in vivo

Lysates from hind limb muscle tissue homogenates harvested at day 21 post-surgery were used for Western blot analysis as described previously[19].Protein was analyzed using 10% sodium dodecyl sulphate-polyacrylamide gel electrophoresis (SDS-PAGE) and transferred to nitrocellulose membranes (Bio-Rad).Membranes were then incubated with primary antibodies including VEGF (1:1000, Cell Signaling) and β-actin (1:5000, Sigma) at 4°C overnight respectively.The membranes were then incubated with peroxidase labeled secondary antibody (1:1000, Santa Cruz, USA) at 37°C for 2 hours. Signals were detected by enhanced chemiluminescence (Amersham, USA). Densitometric analysis for the blots was performed with NIH image software.

### Effect of simvastatin on the cell viability of bone marrow-derived MSCs in vitro

To examine whether simvastatin has anti-apoptotic effect on bone marrow-derived MSCs under hypoxia stress, cells viability was detected by MTT assay and flow cytometry measurement, respectively. Cells (1 × 10^4 ^cells) were cultured in serum-free medium for 24 h with 0.01 μmol/L of simvastatin, 0.01 μmol/L of simvastatin plus 50 n M of wortmannin (phosphatidylinositol 3-kinase, PI3-K, inhibitor), or blank control. Cell viability was evaluated using the MTT assay (MTT, Sigma) and flow cytometry (Becton Dickinson) according to the manufacturer's instructions.

### Effect of simvastatin on the release of VEGF of bone marrow-derived MSCs in vitro

To examine whether simvastatin enhance the release of VEGF by bone marrow-derived MSCs, a total of 1 × 10^4 ^MSCs were plated in serum-free medium with different doses of simvastatin (0, 0.001, 0.01, 0.1 and 1.0 μmol/L) on 48-well plates. VEGF levels in conditioned medium were measured with VEGF ELISA kits (R&D Systems) 24 h after treatment.

### Statistical analysis

All values were expressed as mean ± SD. Student's unpaired *t *test was used to compare differences between every two groups. Comparisons of parameters among three or four groups were made by one-way ANOVA, followed by Scheffe' multiple comparison test. Comparisons of the time course of the LDPI index were made by 2-way ANOVA for repeated measures, followed by Scheffe' multiple comparison tests. A probability value < 0.05 was considered statistically significant.

## Results

### Identification of bone marrow-derived MSCs

**Figure 1:**
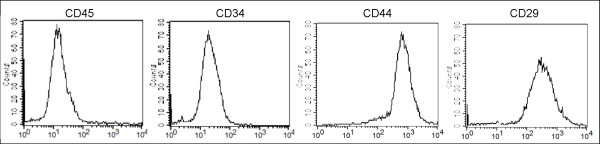
**Characterization of bone marrow-derived MSCs**. Flow cytometric analyses of bone marrow-derived MSCs. Cells were uniformly negative for CD34, CD45, and positive for CD44, and CD29.

During the primary cell culture, the attached cells stretched and took the shape of a typical spindle-shaped fibroblast phenotype. These adherent cells could be readily expanded in vitro by successive cycles of trypsinization, seeding and culture every 3 days for 15 passages without visible morphologic change. Flow cytometry examination showed that these cells were negative for CD34 and CD45, but positive for CD44 and CD29 (Fig. 1). Thus, we designated these fibroblasts-like cell populations as MSCs.

### Combination therapy increases blood perfusion

**Figure 2:**
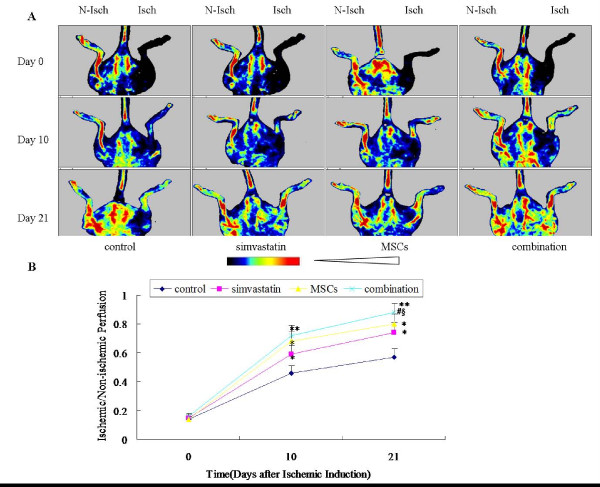
**Effect of simvastatin and bone marrow-derived MSCs administration on the blood reperfusion in ischemic limb**. **A**. In color-coded images, normal perfusion is displayed as red, while low or absent perfusion is displayed as dark blue. Isch = Ischemic limb.N-Isch = Non-Ischemic limb. **B**. Quantitative evaluation of ischemic/normal leg blood perfusion ratio. Values are presented as means ± SD (n = 10). *p < 0.05 and **p < 0.01 versus control, #*p *< 0.05 versus simvastatin, § p < 0.05 versus MSCs.

To determine whether simvastatin or MSCs treatment could stimulate the blood reperfusion in ischemic limb, mice were treated with simvastatin or MSCs or vehicle, the blood reperfusion was examined at day 0, 10 and 21 after the treatment by LDPI. LDPI showed that blood flow in the ischemic hindlimb was decreased equally in all four groups immediately after surgery. Over the subsequent 21 days, blood perfusion of the ischemic hindlimb notably improved in the treatment groups (Fig. 2A) The laser Doppler perfusion index was significantly higher in the simvastatin group, the MSCs group and the combination group than in the control group on day 10 after treatment and showed further improvement afterwards on day 21. The LDPI index was the highest in the combination group among the four groups (Fig. 2B). The normal value of LDPI index was 1.00 ± 0.03 in this study.

### Combination therapy increases capillary density in the ischemic tissues

**Figure 3:**
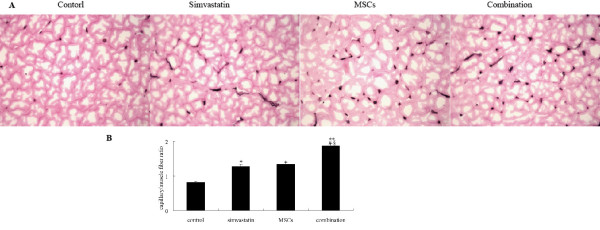
**Effect of simvastatin and bone marrow-derived MSCs administration on angiogenesis in ischemic limb**. **A**. Representative microphotographs of the section of ischemic hindlimb muscles stained histochemically for alkaline phosphatase, magnification × 400. **B**. Quantitative analysis of capillary density in ischemic hindlimb muscles. Data are presented as mean ± SD (n = 10). * *p *< 0.05 and** *p *< 0.01 versus control. # *p *< 0.05 versus simvastatin. § *p *< 0.05 versus MSCs.

To determine whether improved limb reperfusion by the simvastatin treatment or MSCs transplantation was linked with increased angiogenesis in vivo, the capillary/muscle fiber ratio was assessed in the ischemic muscle at 3 weeks after the surgery by histochemical staining for alkaline phosphatase and image analysis (Fig. 3).The number of capillaries in each muscle fiber increased in the mice treated with either MSCs or simvastatin alone in comparison with the control group (*p *< 0.05). The combined administration of simvastatin and MSCs resulted in the highest capillary density (*p *< 0.05 vs. all other groups).

### Combination therapy enhances the differention of MSCs into endothelial cells in ischemic muscles

**Figure 4:**
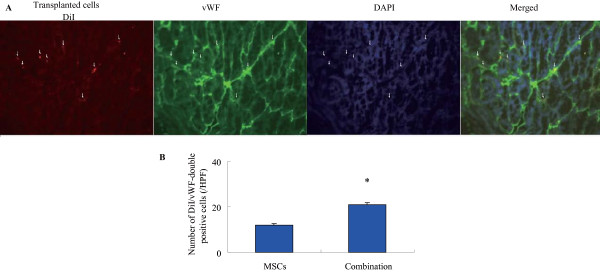
**Effect of simvastatin on differentiation into endothelial cell of MSCs in ischemic limb**. **A**. Representative microphotographs of the section of ischemic hindlimb muscles stained with DiI and immunofluorescence for vWF. Red fluorescence (DiI)-labeled MSCs were transplanted into ischemic thigh muscle and positive of vWF (white arrows indicated). **B**. Quantitative data for the number of DiI/vWF double-positive cells.*p < 0.05 versus MSCs.

To determine whether improved limb reperfusion by simvastatin and MSCs co-therapy was associated with differentiation into endothelial cells, the number of incorporated DiI-labeled MSCs (red labeling) into the mouse microvascular was detected by fluorescent staining against vWF (green labeling) (Fig. 4). Histological and quantitative analyses showed that the number of incorporated MSCs was significantly greater in the combination group relative to MSCs alone (*p *< 0.05).

### Combination therapy decreases cell apoptosis in vivo

**Figure 5:**
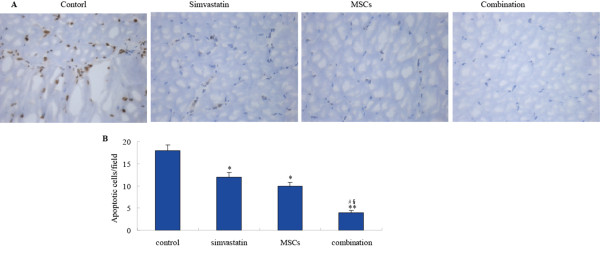
**Effect of simvastatin and bone marrow-derived MSCs on the muscle cells apoptosis in ischemic limb**. **A**. Representative microphotographs of the section of ischemic hindlimb muscles stained immunochemically for TUNEL (brown yellow), magnification × 400. ***B***. Quantitative analysis of apoptosis cells in ischemic hindlimb muscles. Data are presented as mean ± SD (n = 10). * *p *< 0.05 and ** *p *< 0.01 versus control. #*p *< 0.05 versus simvastatin, § *p *< 0.05 versus MSCs.

To determine whether improved limb reperfusion by the simvastatin/MSCs treatment was associated with increased ischemic muscle cells survival in vivo, the cell apoptosis was assessed in the ischemic muscle at days 21 after the treatment by TUNEL assay. Apoptosis as measured by TUNEL positive nuclei (Fig. 5) was significantly decreased in ischemic muscle of simvastatin and MSCs treated mice versus vehicle-treated mice. The co-treatment of simvastatin and MSCs resulted in a further decrease of cell apoptosis.

### Combination therapy enhances the expression of VEGF protein in ischemic tissue

**Figure 6:**
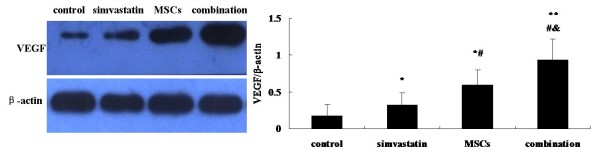
**Effect of simvastatin and bone marrow-derived MSCs on the expression of VEGF in ischemic limb**. The levels of VEGF in ischemic limb 21 days after surgery is measured by Western blot. β-actin was used as an invariant control. *p < 0.05 and **p < 0.01 versus control. #*p *< 0.05 versus simvastatin. &*p *< 0.05 versus MSCs.

To examine whether high-dose simvastatin and MSCs co-therapy improved postischemic neovascularization, the expression of VEGF protein was detected by western blot assay. As can be seen in figure 6, the expression of VEGF significantly increased in the simvastatin group than in the control group (*p *< 0.05). Moreover, the expression of VEGF was higher in MSCs group compared with that in the simvastatin group, but was lower than that in the combination group (*p *< 0.05).

### Effect of simvastatin on the cell viability of bone marrow -derived MSCs in vitro

**Figure 7:**
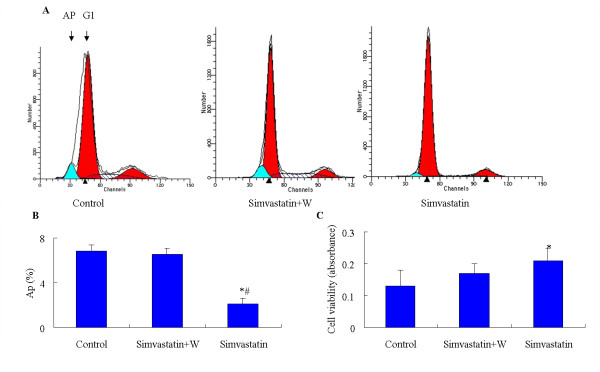
**Effect of simvastatin on the serum-free induced cell viability of bone marrow -derived MSCs in vitro**. Cells were incubated with vehicle or simvastatin plus wortmannin (W), a PI3-K inhibitor, or simvastatin alone for 24 h prior to viability assessment. Wortmannin was added 0.5 h prior to simvastatin. **A**. Histographic representation of nuclear DNA contents measured by flow cytometry. AP = Apoptotic cells. **B**. The percentage of apoptotic MSCs analyzed by flow cytometry. **C**. Cell viability analyzed by MTT. Means ± SD. n = 6 wells per group. The data are representative of 3 individual experiments. * *p *< 0.05 versus control group. # *p *< 0.05 versus simvastatin + W group.

In vitro, serum starvation induced bone marrow-derived MSCs apoptosis, as indicated by flow cytometry and MTT assay. When incubated with 0.01 μmol/L of simvastatin, the percentage of apoptotic cells decreased and the viability was visibly upregulated. However, pretreatment with 50 n M wortmannin, a PI3-K inhibitor, diminished the anti-apoptotic effect of simvastatin (Fig. 7). The cell viability detected by MTT assay was significantly higher in simvastatin treated group than that in the control group. Although the cell viabilities were higher in simvastatin + wortmannin group than those in the control group, there was no significant difference between the two groups. These results indicated that PI3-K pathway was of importance for the anti-apoptotic role of simvastatin.

### Effect of simvastatin on the VEGF releasing of bone marrow -derived MSCs in vitro

**Figure 8:**
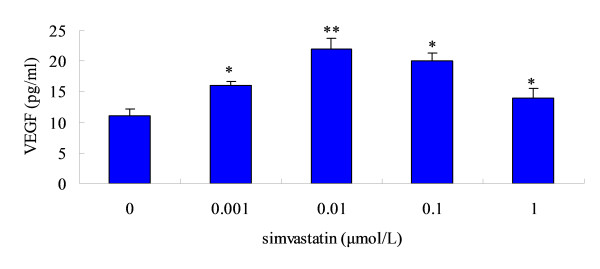
**Effect of simvastatin on the release of VEGF by bone marrow-derived MSCs in vitro**. MSCs were exposed to increasing doses of simvastatin for 24 h. Then, 24 h after replacement of the culture medium, VEGF concentration was measured by ELISA. Means ± SD of measurements from one experiment (n = 4). * *p *< 0.05 versus untreated controls.

To assess whether simvastatin affected the release of VEGF by MSCs, cells were cultured in the absence or presence of various concentration of simvastatin for 24 h and VEGF levels were measured in the conditioned medium using ELISA. Results found that VEGF levels were increased significantly in simvastatin-treated MSC cultures with a maximal effect at 0.01 μmol/L (78.1 ± 5.4 pg/ml) compared to control cultures (38.6 ± 2.2 pg/ml, *p *< 0.05). The VEGF levels were reduced in 0.1 and 1.0 μmol/L simvastatin-treated MSCs cultures when compared with 0.01 μmol/L simvastatin-treated cultures (Fig. 8).

## Discussion

The present study was designed to examine whether high-dose simvastatin could enhance the therapeutic effects of bone marrow-derived stem cells in the treatment of ischemic hindlimb. Overall, this study demonstrates more pronounced angiogenic response, decreased muscle cell apoptosis and improved blood reperfusion of ischemic muscle following combined therapy of high-dose simvastatin and bone marrow-derived MSCs.

Bone marrow-derived MSCs have been identified as a potential new therapeutic option to induce therapeutic angiogenesis. The main advantage of using bone marrow-derived MSCs in treating ischemic disease is that they can be isolated from bone marrow by aspiration and expanded ex vivo before implantation. Under specialized culture conditions, bone marrow-derived MSCs have the capacity to differentiate into cells such as bone, cartilage, adipocytes and endothelial cells [5,20,21]. These results suggest that bone marrow-derived MSCs may be good candidates for cell transplantation. However, some patients are refractory to this cell therapy. Patients with PAD often accompany with several cardiovascular risk factors, such as aging, smoking, diabetes mellitus, et al, which impair the stem cell functions to varying degrees, likely limiting the efficiency of stem cell therapy. Therefore an approach to augment the angiogenic potency of bone marrow-derived MSCs transplantation is of great importance.

Statins, also known as the 3-hydroxy-3-methylglutaryl coenzyme A reductase inhibitors, are the first-line agents used in hypercholesterolemia[19]. They are also characterized by having other benefits apart from their lipid-lowering effects[22]. Among these pleiotropic effects are the anti-apoptotic and pro-angiogenic properties of statins. It has been demonstrated that statins could protect against ischemic injury of the heart and stimulate angiogenesis in ischemic limbs of normocholesterolemic animals. In the present study, we demonstrated that combination of MSCs transplantation and high-dose simvastatin treatment provided advanced benefits on treatment for hindlimb ischemic, compared with either treatment alone.

Combination treatment significantly enhanced capillary density in ischemic limbs than cell therapy alone. This may be partially due to the enhanced stem cells survival and greater differentiation rate of MSCs into vascular cells when administrated with simvastatin simultaneously. In vitro, a higher cell proliferation and decreased apoptosis under serum-free culture was demonstrated after bone marrow-derived MSCs incubated with simvastatin by MTT assay and flow cytometry measurement, respectively. An earlier study has demonstrated that ischemia and mechanical stress induce apoptosis of transplanted bone marrow cells [23]. In this in vitro study, we revealed that simvastatin inhibited serum starvation-induced MSC apoptosis, which may partly be blocked by wortmannin, a PI3-K inhibitor, indicating that the PI-3K/Akt pathway may be an important way in the regulation of MSCs apoptosis.

Enhanced expression of angiogenic growth factors in the ischemic tissue is another contributor to augment angiogenesis resulting from combinatorial treatment. It is well known [24-26] that bone marrow-derived MSCs could paracrine several angiogenic growth factors such as VEGF, etc. On the other hand, recent studies have demonstrated that statins strongly induced angiogenesis with increases in angiogenic cytokines [27,23]. In vitro, a higher expression of VEGF was detected in bone marrow-derived MSCs culture medium compared with blank control, indicating that MSCs could release a mount of angiogenic growth factors in hypoxic environment. Simultaneously, a highest expression of VEGF protein was detected in combination group in vivo. However, the release of VEGF by MSCs was reduced when the concentration of simvastatin increased in vitro. This indicated that the simvastatin has a biphasic dose-dependent effect on angiogenesis in vitro. Weis et al previously demonstrated that statins have proangiogenic effects at low therapeutic concentrations (0.5 mg/kg/d of cerivastatin) but angiostatic effects at high concentrations (2.5 mg/kg/d) in apolipoprotein E-deficient hypercholesterolemic C57BL/6J mice. In the present study, simvastatin augmented angiogenesis in response to acute ischemia at even a higher dose (20 mg/kg/d). Masataka Sata has previously demonstrated [13] that high-dose statins (5 mg/kg/d cerivastatin) promoted blood flow recovery in the ischemic hind limb as determined by LDPI. In a stroke model in mice, atorvastatin (10 mg/kg/d) administered subcutaneously after stroke for 14 days brought about an improvement in neurologic recovery, which was related with an increase in VEGF, VEGF receptor 2, brain-derived neurotrophic factor, and endothelial cell proliferation in the ischemic territory [28]. A recent study revealed [29] that the same dose of simvastatin promoted angiogenesis in response to hypoxic conditions, but decreased angiogenesis mediated by inflammation. Therefore, it might be plausible that proangiogenic or antiangiogenic effects of statins might depend on distinct mechanisms of angiogenesis associated with inflammation, hypoxia, tissue ischemia, or cancer.

Promotion of limb muscle cells survival under hypoxic circumstance might be another contributor to improved blood reperfusion of ischemic muscle following combined therapy of simvastatin and bone marrow-derived MSCs. Apoptosis is defined as a programmed cell death or cell suicide, which determines the lifespan and coordinates the removal of cells. A line of evidence has been demonstrated[30,31]that simvastatin and bone marrow-derived MSCs both have the anti-apoptotic effects. Franke C has demonstrated [30] that simvastatin could upregulate the bcl-2 expression and enchance cells survival. Kortesidis and colleagues also demonstrated[31] that bone marrow derived-MSCs express factors that support cell survival and avoid apoptosis thereby preserving cells which would otherwise be destroyed. As indicated by TUNEL assay (Fig. 5), hindlimb muscle cells underwent severe ischemic apoptosis after artery occlusion. However, the apoptosis cells in ischemic muscle regions were significantly reduced after simvastatin and bone marrow-derived MSCs combined treatment.

Therefore, our study clearly demonstrated that bone marrow-derived MSCs in combination with high-dose simvastatin may be more effective or beneficial during the ischemic scenario than bone marrow-derived MSCs alone.

## Competing interests

The authors declare that they have no competing interests.

## Authors' contributions

YL, DZ and YZ carried out the main experiment and drafted the manuscript. FZ and DZ conceived of the study and designed the experiment. GH helped to finish animal model and finished statistical analysis. All authors read and approved the final manuscript.

## References

[B1] Diehm C Allenberg JR Pittrow D Mahn M Tepohl G Haberl RL Darius H Burghaus I Trampisch HJ Mortality and vascular morbidity in older adults with asymptomatic versus symptomatic peripheral artery disease Circulation 2009 120 2053 2061 10.1161/CIRCULATIONAHA.109.865600 19901192

[B2] Monreal M Alvarez L Vilaseca B Coll R Suarez C Toril J Sanclemente C Clinical outcome in patients with peripheral artery disease. Results from a prospective registry (FRENA) Eur J Intern Med 2008 19 192 197 10.1016/j.ejim.2007.09.003 18395163

[B3] Pittenger MF Mackay AM Beck SC Jaiswal RK Douglas R Mosca JD Moorman MA Simonetti DW Craig S Marshak DR Multilineage potential of adult human mesenchymal stem cells Science 1999 284 143 147 10.1126/science.284.5411.143 10102814

[B4] Dimmeler S Leri A Aging and disease as modifiers of efficacy of cell therapy Circ Res 2008 102 1319 1330 10.1161/CIRCRESAHA.108.175943 18535269 PMC2728476

[B5] MacKenzie TC Flake AW Human mesenchymal stem cells: insights from a surrogate in vivo assay system Cells Tissues Organs 2002 171 90 95 10.1159/000057694 12021494

[B6] Urbich C Dimmeler S Risk factors for coronary artery disease, circulating endothelial progenitor cells, and the role of HMG-CoA reductase inhibitors Kidney Int 2005 67 1672 1676 10.1111/j.1523-1755.2005.00261.x 15840010

[B7] Kalinowski L Dobrucki LW Brovkovych V Malinski T Increased nitric oxide bioavailability in endothelial cells contributes to the pleiotropic effect of cerivastatin Circulation 2002 105 933 938 10.1161/hc0802.104283 11864921

[B8] Haendeler J Hoffmann J Zeiher AM Dimmeler S Antioxidant effects of statins via S-nitrosylation and activation of thioredoxin in endothelial cells: a novel vasculoprotective function of statins Circulation 2004 110 856 861 10.1161/01.CIR.0000138743.09012.93 15289372

[B9] Satoh K Takaguri A Itagaki M Kano S Ichihara K Effects of rosuvastatin and pitavastatin on ischemia-induced myocardial stunning in dogs J Pharmacol Sci 2008 106 593 599 10.1254/jphs.08017FP 18403900

[B10] Matsumura M Fukuda N Kobayashi N Umezawa H Takasaka A Matsumoto T Yao EH Ueno T Negishi N Effects of atorvastatin on angiogenesis in hindlimb ischemia and endothelial progenitor cell formation in rats J Atheroscler Thromb 2009 16 319 326 19672036 10.5551/jat.no026

[B11] Weis M Heeschen C Glassford AJ Cooke JP Statins have biphasic effects on angiogenesis Circulation 2002 105 739 745 10.1161/hc0602.103393 11839631

[B12] Yang YJ Qian HY Huang J Li JJ Gao RL Dou KF Yang GS Willerson JT Geng YJ Combined therapy with simvastatin and bone marrow-derived mesenchymal stem cells increases benefits in infarcted swine hearts Arterioscler Thromb Vasc Biol 2009 29 2076 2082 10.1161/ATVBAHA.109.189662 19762786

[B13] Sata M Nishimatsu H Osuga J Tanaka K Ishizaka N Ishibashi S Hirata Y Nagai R Statins augment collateral growth in response to ischemia but they do not promote cancer and atherosclerosis Hypertension 2004 43 1214 1220 10.1161/01.HYP.0000126186.29571.41 15166180

[B14] Jiang Y Jahagirdar BN Reinhardt RL Schwartz RE Keene CD Ortiz-Gonzalez XR Reyes M Lenvik T Lund T Blackstad M Du J Aldrich S Lisberg A Low WC Largaespada DA Verfaillie CM Pluripotency of mesenchymal stem cells derived from adult marrow Nature 2002 418 41 49 10.1038/nature00870 12077603

[B15] Hu Z Zhang F Yang Z Yang N Zhang D Zhang J Cao K Combination of simvastatin administration and EPC transplantation enhances angiogenesis and protects against apoptosis for hindlimb ischemia J Biomed Sci 2008 15 509 517 10.1007/s11373-008-9243-1 18327656

[B16] Couffinhal T Silver M Zheng LP Kearney M Witzenbichler B Isner JM Mouse model of angiogenesis Am J Pathol 1998 152 1667 1679 9626071 PMC1858441

[B17] Yao Y Zhang F Wang L Zhang G Wang Z Chen J Gao X Lipopolysaccharide preconditioning enhances the efficacy of mesenchymal stem cells transplantation in a rat model of acute myocardial infarction J Biomed Sci 2009 16 74 10.1186/1423-0127-16-74 19691857 PMC2739513

[B18] Tan Y Shao H Eton D Yang Z Alonso-Diaz L Zhang H Schulick A Livingstone AS Yu H Stromal cell-derived factor-1 enhances pro-angiogenic effect of granulocyte-colony stimulating factor Cardiovasc Res 2007 73 823 832 10.1016/j.cardiores.2006.12.015 17258698 PMC2243257

[B19] Sadowitz B Maier KG Gahtan V Basic science review: Statin therapy--Part I: The pleiotropic effects of statins in cardiovascular disease Vasc Endovascular Surg 2010 44 241 251 10.1177/1538574410362922 20403949

[B20] Tang J Xie Q Pan G Wang J Wang M Mesenchymal stem cells participate in angiogenesis and improve heart function in rat model of myocardial ischemia with reperfusion Eur J Cardiothorac Surg 2006 30 353 61 10.1016/j.ejcts.2006.02.070 16829080

[B21] Zimmet JM Hare JM Emerging role for bone marrow derived mesenchymal stem cells in myocardial regenerative therapy Basic Res Cardiol 2005 100 471 481 10.1007/s00395-005-0553-4 16237508

[B22] Futterman LG Lemberg L Statin pleiotropy: fact or fiction? Am J Crit Care 2004 13 244 249 15149060

[B23] Bitto A Minutoli L Altavilla D Polito F Fiumara T Marini H Galeano M Calo M Lo Cascio P Bonaiuto M Migliorato A Caputi AP Squadrito F Simvastatin enhances VEGF production and ameliorates impaired wound healing in experimental diabetes Pharmacol Res 2008 57 159 169 10.1016/j.phrs.2008.01.005 18316203

[B24] Cheng AS Yau TM Paracrine effects of cell transplantation: strategies to augment the efficacy of cell therapies Semin Thorac Cardiovasc Surg 2008 20 94 101 10.1053/j.semtcvs.2008.04.003 18707640

[B25] Chen L Tredget EE Wu PY Wu Y Paracrine factors of mesenchymal stem cells recruit macrophages and endothelial lineage cells and enhance wound healing PLoS One 2008 3 e1886 10.1371/journal.pone.0001886 18382669 PMC2270908

[B26] Gnecchi M He H Noiseux N Liang OD Zhang L Morello F Mu H Melo LG Pratt RE Ingwall JS Dzau VJ Evidence supporting paracrine hypothesis for Akt-modified mesenchymal stem cell-mediated cardiac protection and functional improvement FASEB J 2006 20 661 669 10.1096/fj.05-5211com 16581974

[B27] Moreno PR Sanz J Fuster V Promoting mechanisms of vascular health: circulating progenitor cells, angiogenesis, and reverse cholesterol transport J Am Coll Cardiol 2009 53 2315 2323 10.1016/j.jacc.2009.02.057 19539140

[B28] Chen J Zhang C Jiang H Li Y Zhang L Robin A Katakowski M Lu M Chopp M Atorvastatin induction of VEGF and BDNF promotes brain plasticity after stroke in mice J Cereb Blood Flow Metab 2005 25 281 290 10.1038/sj.jcbfm.9600034 15678129 PMC2804085

[B29] Zhu XY Daghini E Chade AR Lavi R Napoli C Lerman A Lerman LO Disparate effects of simvastatin on angiogenesis during hypoxia and inflammation Life Sci 2008 83 801 809 10.1016/j.lfs.2008.09.029 18976673 PMC2596882

[B30] Franke C Noldner M Abdel-Kader R Johnson-Anuna LN Gibson Wood W Muller WE Eckert GP Bcl-2 upregulation and neuroprotection in guinea pig brain following chronic simvastatin treatment Neurobiol Dis 2007 25 438 445 10.1016/j.nbd.2006.10.004 17157514

[B31] Kortesidis A Zannettino A Isenmann S Shi S Lapidot T Gronthos S Stromal-derived factor-1 promotes the growth, survival, and development of human bone marrow stromal stem cells Blood 2005 105 3793 3801 10.1182/blood-2004-11-4349 15677562

